# How relevant are social costs in economic evaluations? The case of Alzheimer’s disease

**DOI:** 10.1007/s10198-019-01087-6

**Published:** 2019-07-24

**Authors:** L. M. Peña-Longobardo, B. Rodríguez-Sánchez, J. Oliva-Moreno, I. Aranda-Reneo, J. López-Bastida

**Affiliations:** 1 Faculty of Social Science and Law, University of Castilla-La Mancha, Talavera de la Reina, Spain; 2 Faculty of Health Science, University of Castilla-La Mancha, Talavera de la Reina, Spain

**Keywords:** Alzheimer’s disease, Economic evaluation, Labour productivity, Informal care, Societal perspective, Social costs, Cost–effectiveness, Cost–utility, I11, I15, I18, H0

## Abstract

**Background:**

The main objective of this study was to analyse how the inclusion (exclusion) of social costs can alter the results and conclusions of economic evaluations in the field of Alzheimer’s disease interventions.

**Methods:**

We designed a systematic review that included economic evaluations in Alzheimer’s disease. The search strategy was launched in 2000 and ran until November 2018. The inclusion criteria were: being an original study published in a scientific journal, being an economic evaluation of any intervention related to Alzheimer’s disease, including social costs (informal care costs and/or productivity losses), being written in English, using QALYs as an outcome for the incremental cost–utility analysis, and separating the results according to the perspective applied.

**Results:**

It was finally included 27 studies and 55 economic evaluations. Around 11% of economic evaluations changed their main conclusions. More precisely, three of them concluded that the new intervention became cost-effective when the societal perspective was considered, whereas when using just the health care payer perspective, the new intervention did not result in a cost–utility ratio below the threshold considered. Nevertheless, the inclusion of social cost can also influence the results, as 37% of the economic evaluations included became the dominant strategy after including social costs when they were already cost-effective in the health care perspective.

**Conclusions:**

Social costs can substantially modify the results of the economic evaluations. Therefore, taking into account social costs in diseases such as Alzheimer’s can be a key element in making decisions about public financing and pricing of health interventions.

## Introduction

The economic evaluation of health care interventions has become in many countries a basic tool for decision-making [1, 2]. In fact, public decision-makers have the difficult task of combining double objectives. On one hand, they should favour their citizens’ access to the therapeutic advances that are being developed continuously and, therefore, implement those interventions, strategies, and policies with promising effects on health. On the other hand, they should also ensure that the irruption of such health innovations and new policies do not compromise the financial sustainability of public health systems [3]. In this context, economic evaluation is a useful tool to be used when establishing an explicit framework, so that they can help decision-makers prioritise health care policies or interventions, which lead to improvements in health, in an efficient way.

In the field of research that focuses on the economic impact of diseases, there is a growing interest in the literature considering non-health care costs (such as informal care and/or productivity losses). In fact, many cost-of-illness studies have already revealed the importance of non-health or social costs for certain diseases such as vascular diseases, dementia, tumours, and mental or rare diseases, without exhausting the list [4–10]. Furthermore, other works have addressed the relevance of the economic impact of the social costs associated with a set of diseases and injuries [11–18]. However, in the methodological guides of economic evaluation of health technologies, despite a strong degree of consensus on those essential technical aspects that must be present in any evaluation [1], there are other aspects that, because they belong to the normative dimension, raise greater controversies. One of them is the type of perspective that should be used in economic evaluations (health care payer, public payer, and societal) that involve, among other considerations, the types of costs that should be included in the analysis.

In this sense, the considerable impact that social costs might have on society has entailed an encouragement of including them in economic evaluations, as the inclusion or exclusion of non-healthcare costs might have a strong impact on cost–effectiveness outcomes. In fact, Krol et al. analysed how the incremental cost–effectiveness ratio (ICER) varied depending on whether productivity costs were included [19]. The main conclusion was that in 83% of the interventions, the ICERs decreased when labour losses were considered. Moreover, in 17% of the economic evaluations, the ICERs switched from positive to negative, becoming the new treatment a cost-saving alternative when such costs were included. Likely, the weight of informal care may be quite relevant. Thus, not considering them can lead to underestimating the real benefit that interventions have on people with certain limitations to carry out their activities of daily living [20]. In brief, for all the aforementioned, not taking into account costs associated with family caregiving or labour losses may impoverish and bias the real economic and social impacts that any intervention has.

This study focused on the case of Alzheimer’s disease (AD). Several reasons justify the selection of such a chronic disease, first because, as a direct consequence of the increase in life expectancy and population ageing, there is a growth of highly prevalent diseases among older people, such as Alzheimer’s. Second, because of the impact on health care and social resources consumed due to such disease. In fact, this is one of the health problems that entail the greatest global burden [9, 21–27], especially in countries where greater efforts have been made to estimate the economic, social impact and a loss of quality-of-life. Finally, the welfare losses caused by this disease are not limited to patients. The people who take care of people suffering from AD also bear important burdens on their health, their working situations, and their social and family relationships [28–31].

The lack of studies that have verified the extent to which the inclusion (or exclusion) of social costs in economic evaluations can imply a significant change in the results of the evaluations and, if so, whether this could alter the conclusions derived from these studies is striking. To our knowledge, only three studies have recently addressed this issue [19, 20, 32], but none of them has focused on the case of such a prevalent and relevant (in terms of economic burden) chronic disease as AD. Therefore, the main objective of this study was to analyse how the inclusion (exclusion) of social costs could alter the results and, especially, the conclusions of the economic evaluations carried out on any intervention for AD during the past 2 decades.

## Methods

### Design and data source

We designed a systematic review of economic evaluations of any interventions in AD. The strategy search was launched in Medline (PubMed), using the following terms: (“Costs and Cost Analysis” OR “cost–effectiveness” OR “cost–utility” OR “cost–benefit” OR “economic evaluation” OR “economic analysis” OR “QALY” OR “quality-adjusted life years”) AND (“Alzheimer Disease” OR “Alzheimer`s disease” OR “dementia”). We used both formal keywords (MeSH terms) and natural keywords in titles or abstracts. To avoid low sensitivity of the strategy search, we used the Cost–Effectiveness Analysis (CEA) Registry from the Tufts University website to identify economic evaluations that could not have been identified in the Medline search. Some previous studies have used such a complementary database, which ensures a more accurate search [33]. More precisely, the CEA Registry uses an algorithm that is also launched in Medline, using the following keywords: QALYs, quality, and cost–utility analysis. All references identified by the algorithm were fully reviewed and only studies that contained an original economic evaluation with a cost–utility analysis were included in the registry. More details about the process are provided elsewhere [34].

We used the ‘Alzheimer’ and ‘dementia’ terms in the Cost–Effectiveness Registry, and we compared the results obtained with the Medline search to avoid duplicated references. However, the studies retrieved from the ‘dementia’ search term were only included if most of the patients enrolled in the study had an AD diagnosis and it was explicitly indicated in the study. Both search strategies were conducted from the beginning of 2000 until November 2018. The inclusion criteria included were: (1) original studies published in a scientific journal, (2) economic evaluations of any intervention related to the disease, (3) social costs (informal care costs and/or productivity losses) in the analysis, (4) publication in English, (5) using quality-adjusted life year (QALYs) as one of the outcomes for the analysis, and (6) provision separately the results of the cost–utility analysis according to the perspective applied.

### Data extraction

After excluding duplicates, the title and the abstract of all references were revised. Following a full-text review, the following information was collected from the references that met the inclusion criteria: authors, year of publication and journal, type of analysis carried out (cost–utility or cost–effectiveness/cost–utility), country, the type of intervention assessed in the economic evaluation (drugs, non-pharmaceutical therapy, diagnostic or screening device, or a medical procedure), the perspective applied by the authors and the threshold assumed for the economic evaluation, discount rates used for costs and outcomes, the time horizon, the currency, the type of sensitivity analysis performed, which costs were included in the economic evaluation, and the incremental costs and health outcomes resulting from the new intervention against the comparator. Microsoft Excel was used to summarise the results from the systematic literature review.

The data extraction was double-blinded and conducted using peer review. Thus, four researchers experienced in the topic performed the first screening (BRS, JOM, LMPL, and IAR). Then, each abstract and study selected was reviewed by four researchers (BRS, JOM, LMPL, and IAR), and the extraction data were performed independently. Three researchers (BRS, IAR, and LMPL) carried out the second revision. Whenever there was a disagreement, the paper was reviewed by a third researcher (JOM and JLB).

### Definition of social costs and perspective

To identify and limit the concepts of health care and social costs, we followed the System of Health Accounts methodology proposed by the Organisation for Economic Co-operation and Development (OECD) in 2000 and revised in 2011, in this work [35]. We included professional long-term care in health care costs. According to *A System of Health Accounts 2011*, revised edition, ‘Total long-term care consists of a range of medical/nursing care services, personal care services and assistance services that are consumed with the primary goal of alleviating pain, and suffering or reducing or managing the deterioration in health status in patients with a degree of long-term dependency’. Thus, the set of health care costs would encompass general practitioner (GP) and specialist visits, outpatient consultations, nursing care, pharmaceuticals, hospitalisations, imaging and laboratory tests, monitoring care, emergency visits, nursing home, community-based care, social services, out-of-pocket health care expenditures, and travelling costs to be treated.

In this study, social costs were defined as the time of informal caregiving or the patient’s loss of productivity due to the illness. The reason for doing so, it is because the majority of guides agreed on the fact that social costs include the following: long-term care services (formal and informal care), out-of-pocket expenditure, and labour productivity loss. Nevertheless, the vast majority of economic evaluations carried out in Alzheimer’s disease do not take into account neither the non-medical consumption nor out-of-pocket expenditure as the weight of such costs within the total economic impact of the disease is quite low in comparison with the weight of the others (informal care and labour productivity loss) [7, 27, 28]. Therefore, we decided to consider those non-healthcare costs with the highest economic impact on disease. For their estimation, there were several methods of valuating informal caregiving time [36–38] or assessment of productivity losses [39]. We also collected the methods that the authors used for estimating these societal costs.

In addition, we considered two perspectives: the health care payer perspective, including only the aforementioned health care costs, and the societal perspective, when social costs would be added to the health care payer perspective. The costs that should be included from the perspective of society would be labour losses and/or informal care time assessment.

## Results

**Fig. 1:**
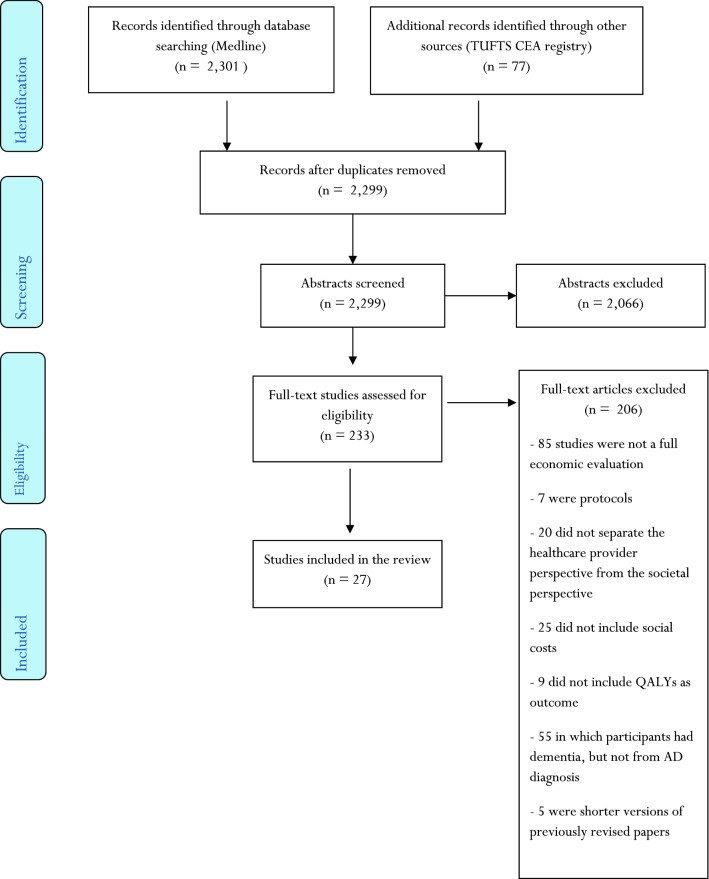
Flowchart of study identification and selection criteria

The search identified a total of 233 studies of interest. After reading all the manuscripts, 206 studies were excluded due to different exclusion criteria (Fig. 1). We identified 72 full cost–utility analyses; 47 of them (65%) included social costs, but only 27 separated both perspectives (health care provider and societal ones). Hence, we selected 27 studies that were full economic evaluations, using QALYs as the main outcome, including social costs (productivity losses or informal caregiving costs or both of them) and with separated health care provider and societal perspectives.

### Study characteristics

From the 27 studies selected [40–66], three of them [47, 51, 57] included both cost–utility analysis (CUA) and Cost–Effectiveness Analysis (CEA), using other outcome measures in addition to QALYs. The remaining 24 studies included only CUA.

Ten studies were made using data from the United Kingdom [41, 43, 47, 48, 51, 55, 57, 59, 60], six from the United States [40, 46, 56, 64–66], two from Spain [45, 51], and another two from France [42, 49]. Other studies used data from Denmark [50], Switzerland [52], Germany [53], Norway [54], Canada [58], The Netherlands [62], and Taiwan [63].

Fifteen studies evaluated a pharmacological intervention [44, 46, 49, 51–55, 58–61, 63–65], whereas another seven studies evaluated a diagnostic or screening procedure [40, 42, 43, 45, 56, 62, 66]; four studies assessed a non-pharmacological therapy [41, 48, 50, 57], and one evaluated a medical procedure [47]. With respect to the perspective used in the studies, most of the studies used both the health care payer and the societal perspective as the main points of view of the analyses [45, 48, 49, 51–56, 58, 60, 61, 63, 64]. Six studies used the societal perspective as the main perspective, but the health care payer perspective could be extracted from the data in the tables [42, 43, 46, 47, 62, 65]. Four studies used the health care provider as the main perspective, but included the societal perspective in the sensitivity analysis [41, 44, 47, 59]. In another three studies, the main perspective was the societal one, with the health care payer perspective included in the sensitivity analysis [40, 50, 66].

The time horizon of the 22 studies widely differed, from 1 year or less [41, 44, 47, 48, 51, 57, 62] to lifetime [40, 43, 56, 65]. Moreover, the discount rate for costs and outcomes varied from 3% [6–66] to a maximum of 5% [58]. The most common sensitivity analysis was the one-way deterministic sensitivity analysis, with some papers also including a probabilistic sensitivity analysis [40, 42, 43, 45, 46, 49, 50, 53, 54, 56, 58–61, 63], a multivariate deterministic sensitivity analysis [45, 49, 53, 54, 58–61], or a scenario analysis [40, 41, 44, 58, 66].

Finally, productivity losses were included only in one study [41]. Out of the 27 studies, 12 [42, 45, 47, 50, 54, 56, 58–62, 66] used the opportunity cost method to calculate informal care costs. Another two studies used the replacement cost method to impute informal caregiving costs [40, 65]. In four more studies, both methods were used [44, 48, 51, 63].

A more detailed description of the characteristics of the selected studies can be found in Table 1.

**Table 1:** Characteristics of the 27 studies selected

Authors and publication year	Type of economic evaluation	Country	Intervention type	Perspective	Discount rate (costs/outcomes)	Time horizon	Costs included	Currency (reference year)	Type of sensitivity analysis	Method to calculate social costs
Michaud et al. (2018) [40]	CUA	United States	Diagnostic/screening	Societal and healthcare payer^a^	3%; 3%	Lifetime	Healthcare costs: tests, medications, office visits due to treatment, community-based care Social costs: informal care	United States Dollar ($)/2016	One-way deterministic and probabilistic sensitivity analyses; scenario analysis	Informal care costs: replacement cost method
Lamb et al. (2018) [41]	CUA	United Kingdom	Non-pharmacological therapy	Healthcare payer and societal^a^	n.a.; n.a.	1 year	Healthcare costs: nursing and personal care, rehabilitation, hospital services, day care services, community care services, mental health care, social services, private health services Social costs: informal care	Sterling Pound (£)/2016	One-way deterministic sensitivity analyses; Scenario analysis	Informal care costs: n.a. Productivity losses: n.a.
Hornberger et al. (2017) [42]	CUA	France	Diagnostic/screening	Societal and healthcare payer^b^	4%; 4%	10 years	Healthcare cost: medications, imaging and laboratory tests, nursing home Social costs: informal care	Euro (€)/2016	One-way deterministic and probabilistic sensitivity analysis	Informal care costs: opportunity cost
Tong et al. (2017) [43]	CUA	United Kingdom	Diagnostic/screening	Societal and healthcare payer^b^	3.5%; 3.5%	Lifetime	Health care cost: GP visits, practise nurse, laboratory tests, social care Social costs: informal care	Sterling Pound (£)/2016	One-way deterministic and probabilistic sensitivity analysis	Informal care costs: n.a.
Knapp et al. (2017) [44]	CUA	United Kingdom	Pharmaceutical	Healthcare payer and societal^a^	N/A; N/A	1 year	Health care cost: hospital care, medications, tests, community-based care Social costs: informal care	Sterling Pound (£)/2014	Societal perspective added	Informal care costs: opportunity cost and replacement cost method
Hornberger et al. (2015) [45]	CUA	Spain	Diagnostic	Societal and healthcare payer	3%; 3%	10 years	Healthcare cost: medications, diagnostic tests, nursing home Social costs: informal care	Euro (€)/2010	One-way and multivariate deterministic; probabilistic sensitivity analysis	Informal care costs: opportunity cost
Saint-Laurent et al. (2015) [46]	CUA	United States	Pharmaceutical	Societal and healthcare payer^b^	3%; 3%	3 years	Healthcare cost: medications, monitoring, medical care Social costs: informal care	United States Dollar ($)/2013	One-way deterministic and probabilistic sensitivity analysis	Informal care costs: n.a.
D’Amico et al. (2015) [47]	CUA/CEA	United Kingdom	Medical procedure	Healthcare payer and societal^a^	3.5%; 3.5%	6 months	Healthcare cost: hospital and day services, equipment and adaptation, medications, social and community care Social costs: informal care	Sterling Pound (£)/2011	One-way deterministic sensitivity analysis	Informal care costs: opportunity cost
Orgeta et al. (2015) [48]	CUA	United Kingdom	Non-pharmacological therapy	Societal and healthcare payer	n.a.; n.a.	1 year	Healthcare cost: nursing and home care, hospital care (inpatient, day, outpatient and accident and emergency services), primary and community health and social care, out-of-pocket payments (travel expenses to health and social care appointments) Social costs: informal care	Sterling Pound (£)/2012	One-way deterministic sensitivity analysis	Informal care costs: opportunity cost and replacement cost method
Touchon et al. (2014) [49]	CUA	France	Pharmaceutical	Societal and healthcare payer	3%; 3%	7 years	Healthcare cost: medications, hospitalizations, medical visits, emergency visits, other medical costs, nursing home care Social costs: informal care	Euro (€)/2013	One-way and multivariate deterministic; probabilistic sensitivity analysis	Informal care costs: n.a.
Sogaard et al. (2014) [50]	CUA	Denmark	Non-pharmacological therapy	Societal and healthcare payer^a^	3%; 3%	3 years	Healthcare cost: hospitalizations, primary care visits, nursing home care Social costs: informal care and productivity losses	Euro (€)/2008	One-way deterministic and probabilistic sensitivity analysis	Informal care costs: opportunity cost
Romeo et al. (2013) [51]	CUA/CEA	United Kingdom	Pharmaceutical	Societal and healthcare payer	N/A; N/A	9 months	Healthcare cost: hospitalizations, primary care visits, social services Social costs: informal care	Sterling Pound (£)/2010	One-way deterministic sensitivity analysis	Informal care costs: opportunity cost and replacement cost method
Pfeil et al. (2012) [52]	CUA	Switzerland	Pharmaceutical	Societal and healthcare payer	3%; 3%	8 years	Healthcare cost: medications, hospitalizations, medical visits, nursing home care Social costs: informal care	Euro (€)/2010	One-way deterministic sensitivity analysis	Informal care costs: n.a.
Hartz et al. (2012) [53]	CUA	Germany	Pharmaceutical	Societal and healthcare payer	3%; 3%	10 years	Healthcare cost: medication; hospitalizations; medical visits, other medical costs, and social services Social costs: informal care	Euro (€)/2008	One-way and multivariate deterministic; probabilistic sensitivity analysis	Informal care costs: n.a.
Rive et al. (2012) [54]	CUA	Norway	Pharmaceutical	Societal and healthcare payer	3%; 3%	5 years	Healthcare cost: medications, hospitalizations, medical visits, emergency visits, and social services Social costs: informal care	Norwegian Krones and Euro (€)/2009	One-way and multivariate deterministic; probabilistic sensitivity analysis	Informal care costs: opportunity cost
Getsios et al. (2012) [55]	CUA	United Kingdom	Pharmaceutical	Societal and healthcare payer	3.5%; 3.5%	10 years	Healthcare cost: visits to GPs and medical doctors, medications, tests, nursing home care Social costs: informal care	Sterling Pound (£)/2007	One-way deterministic sensitivity analysis	Informal care costs: opportunity cost
Guo et al. (2012) [56]	CUA	United States	Diagnostic/screening	Societal and healthcare payer	3%; 3%	Lifetime	Healthcare cost: medications, medical care, social services Social costs: informal care	United States Dollar ($)/2011	One-way deterministic and probabilistic sensitivity analysis	Informal care costs: n.a.
Woods et al. (2012) [57]	CUA/CEA	United Kingdom	Non-pharmacological therapy	Societal and healthcare payer^b^	n.a.; n.a.	10 months	Healthcare cost: nursing care, GP, health visitor, community psychiatrist, psychologist, counsellor, physiotherapist, occupational therapist, care manager, social worker, home-care worker, care attendant, family support worker, dietician Social costs: informal care	Sterling Pound (£)/2010	Scenario analysis	Informal care costs: n.a.
Lachaine et al. (2011) [58]	CUA	Canada	Pharmaceutical	Societal and healthcare payer	5%; 5%	7 years	Healthcare cost: medications, other medical costs, social services Social costs: informal care	Canadian Dollar ($)/2010	One-way and multivariate deterministic; probabilistic sensitivity analysis	Informal care costs: opportunity cost
Nagy et al. (2011) [59]	CUA	United Kingdom	Pharmaceutical	Healthcare payer and societal^a^	3.5%; 3.5%	5 years	Healthcare cost: medications, outpatient visits costs, nursing home care, standard community care Social costs: informal care	Sterling Pound (£)/2008	One-way and multivariate deterministic; probabilistic sensitivity analysis	Informal care costs: opportunity cost
Getsios et al. (2010) [60]	CUA	United Kingdom	Pharmaceutical	Societal and healthcare payer	3.5%; 3.5%	5 years	Healthcare cost: medications, other medical care and social care Social costs: informal care	Sterling Pound (£)/2007	One-way and multivariate deterministic; probabilistic sensitivity analysis	Informal care costs: opportunity cost
López-Bastida et al. (2009) [61]	CUA	Spain	Pharmaceutical	Societal and healthcare payer	3%; 3%	30 months	Healthcare cost: medications, hospitalizations, medical visits, emergency visits Social costs: informal care	Euro (€)/2006	One-way and multivariate deterministic; probabilistic sensitivity analysis	Informal care costs: opportunity cost
Wolfs et al. (2009) [62]	CUA	The Netherlands	Diagnostic	Societal and healthcare payer^b^	N/A; N/A	1 year	Healthcare cost: hospital care, medications, nursing home care, home care, out-of-pocket expenditures, travelling costs Social costs: informal care	Euro (€)/2005	One-way deterministic sensitivity analysis	Informal care costs: opportunity cost
Fuh et al. (2008) [63]	CUA	Taiwan	Pharmaceutical	Societal and healthcare payer	3%; 3%	6 years	Healthcare cost: medications, other medical care Social costs: informal care	United States Dollar ($)/2006	One-way and multivariate deterministic; probabilistic sensitivity analysis	Informal care costs: opportunity cost and replacement cost method
Kirbach et al. (2008) [64]	CUA	United States	Pharmaceutical	Societal and healthcare payer	3%; 3%	13 years	Healthcare cost: physician visits, medications; outpatient and inpatient hospital care Social costs: informal care	United States Dollar ($)/2006	Multivariate deterministic sensitivity analysis	Informal care costs: n.a.
Weycker et al. (2007) [65]	CUA	United States	Pharmaceutical	Societal and healthcare payer^b^	3%; 3%	Lifetime	Healthcare cost: medications, hospitalizations, social services Social costs: informal care	United States Dollar ($)/2005	Multivariate deterministic sensitivity analysis	Informal care costs: replacement cost method
McMahon et al. (2000) [66]	CUA	United States	Diagnostic/screening	Societal and healthcare payer^a^	3%; 3%	18 months	Healthcare cost: drug costs and two-follow-up visits, laboratory and diagnostic tests Social costs: informal care	United States Dollar ($)/1998	One-way deterministic sensitivity analysis; Scenario analysis	Informal care costs: opportunity cost

*CUA* cost–utility analysis, *CEA* cost–effectiveness analysis, *GP* general practitioner, *N/A* not available

^a^The perspective of the analysis could be extracted from tables or the main text

^b^The perspective of the analysis could be extracted from the sensitivity evaluation

### Economic evaluations results

**Fig. 2:**
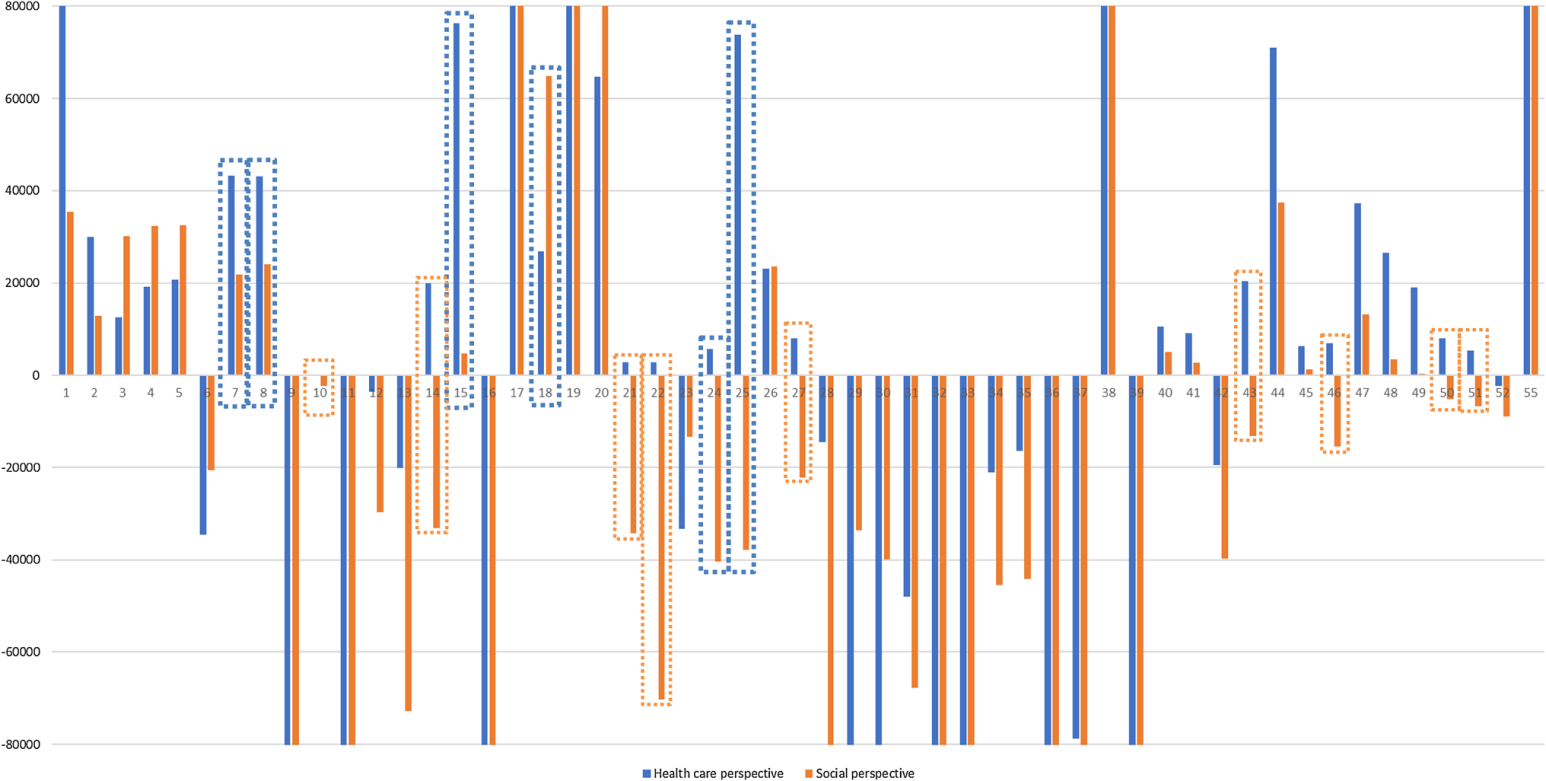
Incremental Cost–Utility Ratios (ICUR) in Alzheimer’s disease interventions: Healthcare and Societal perspectives. *Note:* estimations within a blue square denote that when social costs are included, the conclusions about the cost–effectiveness of the intervention change. Estimations within an orange square mean that if social costs are considered, incremental costs switch from positive incremental costs to negative incremental costs. Estimations number 53 and 54 are not included in this figure, because it was not possible to obtain the ICUR value

From the 27 selected studies, 55 interventions were found in which full economic evaluations were applied. Most papers reported more than one economic evaluation, but 12 of the selected studies included a single result [41, 45, 46, 49, 52, 54, 57, 58, 60, 62–64]. Table 2 and Fig. 2 summarise the results from the 55 full economic evaluations, comparing the conclusions obtained when the health care payer and the societal perspective were considered.

**Table 2:** Results from full economic evaluations on Alzheimer’s disease

Number of estimation		Healthcare payer perspective				Societal perspective				Perspectives comparison	Threshold value
	Authors and publication year	∆Costs	∆QALYs	ICUR (Cost/QALY)	Authors’ conclusions	∆Costs	∆QALYs	ICUR (Cost/QALY)	Authors’ conclusions	Do the conclusions change? (YES/NO)	
1	Michaud et al. (2018) [40]	354	0.004	88,500	Test and treat high or intermediate risk presents a good cost–utility ratio that testing and treating high risk and, hence, it is more cost-effective	142	0.004	35,500	Test and treat high or intermediate risk presents a good cost–utility ratio that testing and treating high risk and, hence, it is more cost-effective	NO	100,000$
2	Michaud et al. (2018) [40]	1137	0.038	29,921	No testing and treating all mild cognitively impaired patients is preferred over testing and treating high-risk patients as no testing and treating is more cost-effective	490	0.038	12,895	No testing and treating all mild cognitively impaired patients is preferred over testing and treating high-risk patients as no testing and treating is more cost-effective	NO	100,000$
3	Michaud et al. (2018) [40]	1953	0.156	12,519	No testing and no mild cognitive impairment treatment has a better cost–utility ratio than testing and treating high-risk patients and, thus, it is more cost-effective	4709	0.156	30,186	No testing and no mild cognitive impairment treatment has a better cost–utility ratio than testing and treating high-risk patients and, thus, it is more cost-effective	NO	100,000$
4	Michaud et al. (2018) [40]	3373	0.176	19,165	Testing and treating low-risk patients is preferred over testing and treating high-risk patients, as testing and treating low-risk patients has a good cost–utility ratio compared to testing and treating high-risk patients	5693	0.176	32,347	Testing and treating low-risk patients are preferred over testing and treating high-risk patients, as testing and treating low-risk patients has a good cost–utility ratio compared to testing and treating high-risk patients	NO	100,000$
5	Michaud et al. (2018) [40]	3726	0.18	20,700	Testing and treating low or intermediate risk patients is more cost-effective than testing and treating high-risk patients as it presents a good cost–utility ratio	5835	0.18	32,417	Testing and treating low or intermediate risk patients is more cost-effective than testing and treating high-risk patients as it presents a good cost–utility ratio	NO	100,000$
6	Lamb et al. (2018) [41]^a^	1347	− 0.039	− 34,538	The exercise therapy is dominated by usual care, as the exercise therapy is more costly and leads to health losses	1301	− 0.063	− 20,651	The exercise therapy is dominated by usual care, as the exercise therapy is more costly and leads to health losses	NO	20,000–30,000£
7	Hornberger et al. (2017) [42]	909	0.021	43,286	Usual care is preferred over florbetapir, as florbetapir does not present a good cost–utility ratio compared with usual care	470	0.021	21,888	Florbetapir presents a good cost–utility ratio and it is preferred over usual care	YES: the new intervention (florbetapir) becomes cost-effective, compared to usual care	40,000€
8	Hornberger et al. (2017) [42]	947	0.022	43,045	Cerebrospinal fluid test is preferred over florbetapir, since florbetapir does not present a good cost–utility ratio	528	0.022	24,084	Florbetapir has a good cost–utility ratio and it is chosen over cerebrospinal fluid test	YES: the new intervention (florbetapir) becomes cost-effective, compared to cerebrospinal fluid test	40,000€
9	Tong et al. (2017) [43]	− 65,755	0.1031	− 637,779	MMSE dominates standard diagnostic tool	− 66,566	0.1031	− 645,645	MMSE dominates standard diagnostic tool	NO	30,000£
10	Tong et al. (2017) [43]	693	3.4847	199	6CIT presents a good cost–utility ratio and it is chosen over standard diagnostic tool	− 7845	3.4847	− 2251	6CIT dominates standard diagnostic tool	NO, but, when social costs are introduced, the new intervention (6CIT) becomes cost-saving and, hence, dominates the comparator (standard diagnostic tool)	30,000£
11	Tong et al. (2017) [43]	− 185,846	0.3063	− 606,745	GPCOG dominates standard diagnostic tool	− 187,064	0.3063	− 610,722	GPCOG dominates standard diagnostic tool	NO	30,000£
12	Knapp et al. (2017) [44]^a^	− 389	0.11	− 3536	Donepezil continuation dominates donepezil discontinuation	− 2669	0.09	− 29,656	Donepezil continuation dominates donepezil discontinuation	NO	20,000–30,000£
13	Knapp et al.(2017) [44]^b^	− 1409	0.07	− 20,129	Memantine dominates memantine placebo	− 1457	0.02	− 72,850	Memantine dominates memantine placebo	NO	20,000–30,000£
14	Knapp et al. (2017) [44]^a^	599	0.03	19,967	Donepezil combined with memantine presents a good cost–utility ratio compared to donepezil alone	− 331	0.01	− 33,100	Donepezil combined with memantine dominates donepezil alone	NO, but, when social costs are introduced, donepezil combined with memantine becomes cost-saving and, hence, dominates the use of donepezil alone	20,000–30,000£
15	Hornberger et al. (2015) [45]	601	0.008	76,268	Florbetapir together with conventional treatment does not have a good cost–utility ratio compared to conventional treatment	36	0.008	4769	Florbetapir together with conventional treatment presents a good cost–utility ratio and it is preferred over conventional treatment	YES: the new intervention (florbetapir and conventional treatment together) becomes cost-effective, compared to conventional treatment	30,000€
16	Saint-Laurent et al. (2015) [46]	− 20,947	0.13	− 161,131	Combination therapy dominates AChEI monotherapy	− 18,355	0.13	− 152,958	Combination therapy dominates AChEI monotherapy	NO	50,000$
17	D’Amico et al. (2015) [47]	475	0.0013	365,276	MCST does not presents a good cost–utility ratio compared to TAU	1146	0.0013	882,801	MCST does not present a good cost–utility ratio compared to TAU	NO	20,000–30,000£
18	D’Amico et al. (2015) [47]	474	0.0176	26,835	MCST does not presents a good cost–utility ratio if threshold is 20,000; MCST has a good cost–utility ratio, compared to TAU, if threshold is 30,000	1143	0.0176	64,842	MCST does not have a good cost–utility ratio, compared to TAU	YES: the new intervention (florbetapir) is no longer cost-effective, compared with usual treatment, after the inclusion of social costs	20,000–30,000£
19	D’Amico et al. (2015) [47]	518	0.0039	132,539	MCST does not have a good cost–utility ratio, compared to TAU	1575	0.0039	400,993	MCST does not have a good cost–utility ratio, compared to TAU	NO	20,000–30,000£
20	D’Amico et al. (2015) [47]	402	0.0062	64,785	MCST does not have a good cost–utility ratio, compared to TAU	1259	0.0062	205,079	MCST does not have a good cost–utility ratio, compared to TAU	NO	20,000–30,000£
21	Orgeta et al. (2015) [48]	140	0.05	2800	Individual cognitive stimulation therapy (iCST) has a good cost–utility ratio, compared with treatment as usual (TAU) and, hence, iCST is more cost-effective than TAU, when using the opportunity cost method to value informal care	− 1710	0.05	− 34,200	Individual cognitive stimulation therapy (iCST) has a good cost–utility ratio, compared with treatment as usual (TAU) and, hence, iCST is more cost-effective than TAU, when using the opportunity cost method to value informal care	NO, but, when social costs are introduced, the new intervention (iCST) becomes cost-saving and, hence, dominates the comparator (TAU)	20,000–30,000£
22	Orgeta et al. (2015) [48]	140	0.05	2800	iCST has a good cost–utility ratio, compared with TAU and, hence, iCST is more cost-effective than TAU, when using the replacement cost method to value informal care	− 3510	0.05	− 70,200	iCST has a good cost–utility ratio, compared with TAU and, hence, iCST is more cost-effective than TAU, when using the replacement cost method to value informal care	NO, but, when social costs are introduced, the new intervention (iCST) becomes cost-saving and, hence, dominates the comparator (TAU)	20,000–30,000£
23	Touchon et al. (2014) [49]	− 8341	0.25	− 33,364	Memantine and ChEI together dominate ChEI as monotherapy	− 3318	0.25	− 13,272	Memantine and ChEI together dominate ChEI as monotherapy	NO	23,000–35,000€
24	Sogaard et al. (2014) [50]^a^	− 957	− 0.17	5629	Psychosocial intervention presents a good cost–utility ratio compared to usual care in complete-case analysis	15,348	− 0.38	− 40,390	Psychosocial intervention is dominated by usual care in complete-case analysis	YES: after including social costs, the psychosocial intervention leads to higher costs and health losses, compared to usual care	100,000€
25	Sogaard et al. (2014) [50]^a^	− 4433	− 0.06	73,883	Psychosocial intervention presents a good cost–utility ratio, compared to usual care, in multiple imputation-based analysis	3401	− 0.09	− 37,789	Psychosocial intervention is dominated by usual care in multiple imputation-based analysis	YES: the new intervention (psychosocial intervention) leads to higher costs and health losses than usual care, after introducing social costs	100,000€
26	Romeo et al. (2013) [51]	693	0.03	23,100	Mirtazapine presents a good cost–utility ratio, compared with placebo	705	0.03	23,500	Mirtazapine presents a good cost–utility ratio, compared with placebo	NO	30,000£
27	Romeo et al. (2013) [51]	404	0.05	8080	Mirtazapine presents a good cost–utility ratio over placebo	− 1106	0.05	− 22,120	Mirtazapine dominates placebo	NO, but, when social costs are included, mirtazapine becomes cost-saving and, hence, dominates placebo	30,000£
28	Romeo et al. (2013) [51]	− 289	0.02	− 14,450	Mirtazapine dominates placebo	− 1811	0.02	− 90,550	Mirtazapine dominates placebo	NO	30,000£
29	Romeo et al. (2013) [51]	− 23,312	0.12	− 194,263	Memantine together with ChEI dominates ChEI as monotherapy	− 4029	0.12	− 33,576	Memantine together with ChEI dominates ChEI as monotherapy	NO	30,000£
30	Pfeil et al. (2012) [52]	− 27,656	0.12	− 230,467	Memantine plus ChEI alternative dominates ChEI as monotherapy	− 4780	0.12	− 39,833	Memantine and ChEI alternative dominates ChEI as monotherapy	NO	100,000 CHF
31	Hartz et al. (2012) [53]	− 7007	0.146	− 47,993	Donepezil dominates no treatment	− 9893	0.146	− 67,760	Donepezil dominates no treatment	NO	N.A.
32	Hartz et al. (2012) [53]	− 1960	0.017	− 115,294	Donepezil dominates memantine	− 2825	0.017	− 166,176	Donepezil dominates memantine	NO	N.A.
33	Rive et al. (2012) [54]	− 47,186	0.03	− 1,572,867	Memantine dominates usual care	− 30,041	0.03	− 1,001,367	Memantine dominates usual care	NO	N.A.
34	Getsios et al. (2012) [55]	− 3593	0.17	− 21,135	Early assessment and treatment with donepezil dominates no early assessment/no treatment	− 7741	0.17	− 45,535	Early assessment and treatment with donepezil dominates no early assessment/no treatment	NO	20,000–30,000£
35	Getsios et al. (2012) [55]	− 2135	0.13	− 16,423	Early assessment and treatment with donepezil dominates treatment without early assessment	− 5726	0.13	− 44,046	Early assessment and treatment with donepezil dominates treatment without early assessment	NO	20,000–30,000£
36	Guo et al. (2012) [56]^b^	− 12,374	0.15	− 82,493	Florbetaben PET dominates usual diagnostic care	− 11,086	0.03	− 369,533	Florbetaben PET dominates usual diagnostic care	NO	50,000$
37	Guo et al. (2012) [56]^b^	− 11,806	0.15	− 78,707	Florbetaben PET dominates usual diagnostic care	− 11,389	0.03	− 379,633	Florbetaben PET dominates usual diagnostic care	NO	50,000$
38	Woods et al. (2012) [57]	1544	0.001	1,544,000	Reminiscence does not have a good cost–utility ratio compared to usual care	2680	0.001	2,680,000	Reminiscence does not have a good cost–utility ratio compared to usual care	NO	N.A.
39	Lachaine et al. (2011) [58]	− 30,512	0.26	− 117,354	Memantine and ChEI dominates ChEI as monotherapy	− 21,391	0.26	− 82,273	Memantine and ChEI dominates ChEI as monotherapy	NO	N.A.
40	Nagy et al. (2011) [59]	1174	0.1109	10,579	Rivastigmine patch presents a good cost–utility ratio compared to best supportive care	− 570	0.1109	5135	Rivastigmine patch dominates best supportive care	NO, but, when social costs are introduced, the new intervention (rivastigmine patch) becomes cost-saving and, hence, dominates the comparator (best supportive care)	20,000£
41	Nagy et al. (2011) [59]	1011	0.1109	9114	Rivastigmine patch presents a good cost–utility ratio compared to best supportive care	301	0.1109	2716	Rivastigmine patch presents a good cost–utility ratio compared to best supportive care	NO	20,000£
42	Getsios et al. (2010) [60]	− 2337	0.12	− 19,475	The donepezil alternative dominates no treatment	− 4769	0.12	− 39,742	The donepezil alternative dominates no treatment	NO	20,000–30,000£
43	López-Bastida et al. (2009) [61]	1971	0.097	20,353	Donepezil presents a good cost–utility ratio over no treatment in mild *AD*	− 1273	0.097	− 13,124	Donepezil dominates no treatment in mild *AD*	NO, but, if social costs are included, donepezil becomes cost-saving and, hence, dominates no treatment in mild AD	25,000–30,000€
44	López-Bastida et al. (2009) [61]	2043	0.029	70,448	Donepezil does not present a good cost–utility ratio over no treatment in *moderate AD*	1080	0.029	37,241	Donepezil does not present a good cost–utility ratio over no treatment in moderate *AD*	NO	25,000–30,000€
45	Wolfs et al. (2009) [62]	316	0.05	6320	Integrated multidisciplinary diagnostic facility presents a good cost–utility ratio over usual care	65	0.05	1267	Integrated multidisciplinary diagnostic facility presents a good cost–utility ratio over usual care	NO	45,000€
46	Fuh et al. (2008) [63]	3677	0.525	7009	Donepezil has a good cost–utility ratio compared to usual care	− 8153	0.525	− 15,529	Donepezil dominates usual care	NO, but, when social costs are introduced, donepezil becomes cost-saving and, hence, dominates usual care	15,000–20,000$
47	Kirbach et al. (2008) [64]	5566	0.15	37,104	Olanzapine presents a good cost–utility ratio compared to no treatment	1985	0.15	13,230	Olanzapine presents a good cost–utility ratio compared to no treatment	NO	50,000$
48	Weycker et al. (2007) [65]	334	0.0126	26,508	Memantine together with donepezil presents a good cost–utility ratio compared to donepezil only at 6 months	44	0.0126	3475	Memantine together with donepezil presents a good cost–utility ratio compared to donepezil only at 6 months	NO	50,000$
49	Weycker et al. (2007) [65]	521	0.0275	18,946	Memantine together with donepezil presents a good cost–utility ratio compared to donepezil only at 12 months	10	0.0275	382	Memantine together with donepezil presents a good cost–utility ratio compared to donepezil only at 12 months	NO	50,000$
50	Weycker et al. (2007) [65]	221	0.0275	8036	Memantine together with donepezil presents a good cost–utility ratio compared to donepezil only at 18 months	− 140	0.0275	− 5102	Memantine together with donepezil dominates donepezil only at 18 months	NO, but, if social costs are introduced, the joint use of memantine and donepezil is cost-saving and, hence, dominates donepezil alone at 18 months	50,000$
51	Weycker et al. (2007) [65]	150	0.0276	5436	Memantine together with donepezil presents a good cost–utility ratio compared to donepezil only at 24 months	− 182	0.0276	− 6613	Memantine together with donepezil dominates donepezil only at 24 months	NO, but, when social costs are introduced, using memantine together with donepezil becomes cost-saving and, hence, dominates the use of donepezil alone at 24 months	50,000$
52	Weycker et al. (2007) [65]	− 62	0.0272	− 2279	Memantine together with donepezil dominates donepezil only during lifetime	− 242	0.0272	− 8897	Memantine together with donepezil dominates donepezil only during lifetime	NO	50,000$
53	McMahon et al. (2000) [66]	N.A.	− 0.0038	Dominated	Visual single-photon emission computed tomography (SPECT) is dominated by standard examination	600	− 0.0038	− 157,895	Visual single-photon emission computed tomography (SPECT) is dominated by standard examination	NO	N.A.
54	McMahon et al. (2000) [66]	N.A.	− 0.0001	Dominated	Computed SPECT is dominated by standard examination	787	− 0.0001	− 7,870,000	Computed SPECT is dominated by standard examination	NO	N.A.
55	McMahon et al. (2000) [66]	691	0.0021	328,830	Magnetic resonance (MR) imaging plus dynamic susceptibility contrast (DSC) MR imaging does not present a good cost–utility ratio, compared to the standard examination	1007	0.0021	479,524	Magnetic resonance (MR) imaging plus dynamic susceptibility contrast (DSC) MR imaging does not present a good cost–utility ratio, compared to the standard examination	NO	N.A.

^a^Differences in health effects when including social costs are due to the inclusion of caregiver’s utility values

^b^Differences in effects change when social costs are included due to the smaller sample with a societal perspective

When the perspective was the one from the health care payer, 21 out of the 55 economic evaluations reported negative incremental costs from the intervention. However, if the societal perspective was considered, 29 economic evaluations incurred negative incremental costs. These differences in the results of negative incremental costs led to a modification of the results in 12 economic evaluations. From those 12 modified results, 10 concluded from the health care payer perspective that the new intervention increased costs, but if a societal perspective was considered, the intervention would lead to cost savings (number of estimations 10, 14, 21, 22, 27, 40, 43, 46, 50, 51). Moreover, the remaining two economic evaluations showed the opposite pattern, leading to cost savings from the health care payer perspective, but with increasing costs from the societal perspective (number of estimations 24 and 25).

The changes in the incremental costs also led to changes in the Incremental Cost–Utility Ratios (ICUR) and, in some cases, to changes in the conclusions, according to the threshold values considered by the authors. From the health care payer perspective, 19 of the 55 economic evaluations reported that the new intervention dominated (negative incremental costs and higher utility values) standard of care or no treatment. Nine positive ICURs, when compared against the threshold used in each article, led to the conclusion that the intervention was not cost-effective and, hence, it was not preferred over the standard of care. From the societal perspective, 29 out of the 55 economic evaluations concluded that the intervention analysed in the study dominated the comparator. Moreover, seven positive ICURs led to the assessed intervention not being preferred over usual care or no treatment.

Hence, when the societal perspective was considered, the results from three economic evaluations were changed with respect to the results obtained from the health care payer perspective, in which the intervention was not cost-effective compared to the standard of care and switched to being cost-effective (number of estimations 7, 8, 15). This change was reported, because, when social costs were included, the new intervention or treatment resulted in lower positive incremental costs. However, three estimations showed the opposite trend. Estimation number 18 reported positive incremental costs, regardless of the perspective considered, but the new intervention showed a good cost–utility ratio compared to the comparator from the health care payer perspective (for a threshold value of 30,000£); nevertheless, if social costs were introduced, the new intervention did not have a good cost–utility ratio. The second and third exceptions (number of estimations 24 and 25) showed that, from the health care perspective, the new intervention led to cost savings and quality-of-life reductions (as the sample changed when societal costs were considered), so the intervention was cost-effective over the standard of care. We also observed that, even if the conclusion did not vary and the assessed intervention would finally be implemented, ten economic evaluations reported that, after considering social costs, the new intervention changed from being cost-effective and showing a good cost–utility ratio to being the dominant strategy (estimations 10, 14, 21, 22, 27, 40, 43, 46, 50, 51). When social costs were included, there were positive incremental costs with quality-of-life reductions too, concluding that the intervention would never be the dominant alternative (number of estimations 6, 24, 25, 53 and 54).

## Discussion

This study analysed how the inclusion of social costs could change the main results and conclusions of economic evaluations of interventions for Alzheimer’s disease. It identified 55 full economic evaluations in which both health care payer and societal perspectives were included, and, therefore, the results of the incremental cost–effectiveness could be analysed separately.

The results showed that only 6 out of these 55 economic evaluations (around 11%) changed their main conclusions when social costs were included. More precisely, in three of them, the new intervention became cost-effective when the societal perspective was considered, contrary to what occurred when using only the health care payer perspective. Namely, in these three economic evaluations whose conclusions changed, and, consequently, the new alternative switched to a cost-effective option, the factor that mainly explained this change was that informal caregiving costs were highly different in the new intervention vs. its comparator. However, the remaining three economic evaluations showed the opposite pattern, leading to cost savings from the health care payer perspective but with increasing costs from the societal perspective.

Such different conclusions might be explained by several factors. First, when the interventions considered were any type of diagnostic tools, the difference in social costs between interventions was very large, whereas when the interventions were medical procedures, such difference was lower. In this sense, it should be stressed that diagnosis interventions have an important effect on the personal care of patients, making family costs considerably lower in comparison with its alternative. Another element that may explain the different results could be the time horizon. In interventions whose time horizon was large (10 years or even more), the difference in social costs was greater than interventions whose time horizon was shorter (fewer than 3 years). This might be because the interventions may have a relevant effect on personal care (that is, the caregiving time), reducing the time required for care, but such effect can be seen only in the long term. Third, the inclusion of costs associated with hospitalisations could also lead to changes in the incremental cost–effectiveness ratio. Congruently, one of the main factors that interventions have an impact on is the number of hospitalisations that such populations have. For this reason, in economic evaluations in which hospitalisation costs were not included, the differences between health care costs were higher than the differences in social costs. Finally, for those countries with a strong cultural aspect in relation to family care (Italy or Spain), the time dedicated to people with Alzheimer’s disease was much higher [10], and, therefore, such a population was prone to receive a higher amount of caregiving time than people in other countries such as in France or northern Europe. Then, the difference in social cost (family caregiving) was higher when the interventions took place in Italy or Spain.

Such results cannot be directly compared with others obtained previously, but a preceding study has already shown that the exclusion of informal caregiving costs in the economic evaluations affects the conclusions [20]. More precisely, the ICER reported by the authors in their systematic review increased from 26,000 to 59,000€ per QALY after the exclusion of costs related to informal caregiving, and it identified that only 23% of economic evaluation included the cost of family care in the evaluation. However, it should be pointed out that Krol et al. did not focus on Alzheimer’s disease and included several types of chronic disease (Alzheimer’s disease, metastatic colorectal cancer, Parkinson’s disease, and rheumatoid arthritis). Nevertheless, in the case of Alzheimer’s disease, out of 25 economic evaluations found, 64% of them (14) included informal care costs, whereas this paper found 55 economic evaluations, in which 65% (35) included social costs (34 evaluations included informal care costs and one evaluation included labour productivity costs). Thus, the main contribution of our study, in comparison with Krol, is that our time horizon was quite a bit wider, from 2000 to 2018, whereas Krol only included the economic evaluations carried out from 2008 to 2013.

It should be noted that the inclusion of social costs does not always benefit the intervention considered innovative. This is especially interesting, because it shows that, in some cases, the intervention implies a transfer of resources from the professional to the family sector, increasing the time of informal care. In this regard, beyond the monetary cost of informal care, it is important to stress the fact that most of the included economic evaluations (86%) did not take into account the effect that the intervention had on the caregivers’ health and well-being (when caregiving time was included in the analysis). This implies, on one hand, that this transfer can place a heavy burden on caregivers and a worsening of their well-being beyond a change in the usual use of time. On the other hand, it had been proven that interventions that improve the health and well-being of the patients with Alzheimer’s could also have an effect on the burden supported by caregivers [29–31], and, consequently, an improvement of the carers’ health status. For these reasons, the inclusion of changes in the health of informal caregivers and the identification of spill-overs of programmes of support to caregivers are aspects of methodological improvement that should be considered in future studies.

Other methodological elements are identified in the reviewed studies that must be mentioned. First, several studies did not mention the discount rate used and whether it was applied to costs and health outcomes. Second, several evaluations did not indicate the method applied to assess the time of informal care. Moreover, even though most of the analysis used the opportunity cost method or proxy good method, in some studies, the unit value of informal care time was not indicated, which makes comparison between studies very difficult. Another methodological element that would help in the comparability between studies would be to include, explicitly, how the time is collected, that is, whether the caregiving time is registered through diaries, the recall method or direct questions, apart from revealing whether any censorship in the daily or weekly caregiving time is applied [9]. Regarding costs associated with productivity losses, it should be highlighted that this type of cost is not commonly applied in studies related to AD, mainly because the average age of such a population is considerably higher, so those people would not be in the labour market. In our case, only 1 out of 55 of the economic evaluations included productivity losses in the analysis.

It is well known that people with Alzheimer’s disease usually require personal care due to the disability and dependence which they suffer from, and, consequently, that the weight of the cost associated with informal care might exceed the health care costs [7, 67, 68]. In fact, 65% of the economic evaluations of Alzheimer’s disease include social costs, especially costs associated with informal care, whereas in other chronic diseases such as metastatic colorectal cancer, Parkinson’s disease, and rheumatoid arthritis, this percentage barely reaches 0%, 13%, and 14%, respectively [20]. Furthermore, in two studies that the authors of this paper are currently carrying out, they have identified that 13% and 17% of economic evaluations of rare diseases and diabetes, respectively, included social costs. Therefore, not taking into account all the resources used in the care of Alzheimer’s patients (professional and non-professional care) and obviating the effects on the health and well-being of non-professional (informal) carers can lead to an underestimation of the real economic impact of this disease. This may cause unintended biases that result in poorly informed decision-making.

Thus, we could find situations in which the implementation of a programme or intervention was adopted if the resource used was family care, but would no longer be considered efficient if this care was replaced by professional care. Logically, this can be consistent if the relevant perspective is that of the health financer of the resources, but would cease to be if the perspective to be applied was a societal one, as recommended by the economic evaluation guides of many countries [1]. In this regard, it is worth noting that the tendency of long-term care systems is evolving in recent years towards a mixed model of shared responsibilities between the family and the state. Therefore, first, it would be expected that in the care of people with AD, except in the case of institutionalisation, both types of care would be provided simultaneously. In this framework, it is not reasonable to value some resources and ignore others in the economic evaluation of health interventions. Second, future projections of spending on long-term care (LTC) coincide with signalling significant growth. However, such projections are based on demographic factors and the evolution of the health status of the population, but there is no evidence on projections about endowments and the future availability of informal care. This implies that informal care could continue to be a socially valuable resource that favours the sustainability of the LTC system, but, at the same time, it could also become a scarcer resource, which would encourage governments to implement programmes that encourage complementarity between both types of care [69, 70]. In any of the cases, the inclusion of all costs (health care, social services, and non-professional care) provides more information to decision-makers and favours analysis of the degree of substitutability or complementarity that exists between these three types of resources. This should lead to the design of more efficient and equitable programmes and interventions.

In brief, despite the importance of the social costs of Alzheimer’s disease, there is still a lack of evidence about the consequences of excluding social costs in economic evaluations in such a population. Societal perspective is habitually recommended in the literature, since it provides comparatively a wider outlook. In fact, national guidelines of many countries recommend the inclusion of societal perespective (Austria, Denmark, France, The Netherland, Norway, Portugal, Spain and Sweden) and both perspectives (Italy, Russia, Slovak Public, Slovenia and Switzerland). Other countries recommend health care funder (USA, Scotland, Poland, Ireland, Germany, Finland, England and Wales, Croatia, Czech Republic, Canada, Belgium, Baltic states and Australia) but in many of them the possibility of including societal perspective in the analysis is comtemplated when being relevant. Broadly, this study contributes to the existing literature by performing an analysis of whether the inclusion of a societal perspective in economic evaluations alters results and conclusions in the specific area of Alzheimer’s disease. Thus, our findings suggest that not considering the social costs of diseases such as Alzheimer’s would lead to materially relevant information not being given to decision-makers. In addition, for the perspective to be truly social, the loss of health due to the overload that informal care can cause should also be considered. The figures shown can illustrate that the adoption of a social perspective can be crucial in making accurate decisions in relation to public financing allocation as well as pricing innovative health interventions in the context of Alzheimer’s disease.
