# Correction to: How relevant are social costs in economic evaluations? The case of Alzheimer’s disease

**DOI:** 10.1007/s10198-021-01323-y

**Published:** 2021-05-25

**Authors:** L. M. Peña-Longobardo, B. Rodríguez-Sánchez, J. Oliva-Moreno, I. Aranda-Reneo, J. López-Bastida

**Affiliations:** 1 Faculty of Social Science and Law, University of Castilla-La Mancha, Talavera de La Reina, Spain; 2 Faculty of Health Science, University of Castilla-La Mancha, Talavera de La Reina, Spain

## Correction to: The European Journal of Health Economics (2019) 20:1207–1236 10.1007/s10198-019-01087-6

The article How relevant are social costs in economic evaluations? The case of Alzheimer’s disease, written by L. M. Peña-Longobardo, B. Rodríguez-Sánchez, J. Oliva-Moreno, I. Aranda-Reneo and J. López-Bastida was originally published electronically on the publisher’s internet portal (currently SpringerLink) on 24 July 2019 without open access. With the author(s)’ decision to opt for open choice, the copyright of the article changed on 20 May 2021 to © The Author(s) 2021 and the article is forthwith distributed under the terms of the Creative Commons Attribution 4.0 International License (http://creativecommons.org/licenses/by/4.0/), which permits use, duplication, adaptation, distribution and reproduction in any medium or format, as long as you give appropriate credit to the original author(s) and the source, provide a link to the Creative Commons license and indicate whether changes were made.

The original article has been corrected.

